# Cardiovascular health in pregnancy according to Life’s Essential 8 score

**DOI:** 10.1038/s44325-026-00117-6

**Published:** 2026-04-01

**Authors:** Krishin Yerabolu, Harshvir S. Bal, Abdulla Shahid, Nehal Vekariya, Naman S. Shetty, Mokshad Gaonkar, Nirav Patel, Peng Li, Pankaj Arora, Garima Arora

**Affiliations:** 1 Division of Cardiovascular Disease, University of Alabama at Birmingham, Birmingham, AL USA; 2 Department of Anesthesia, Critical Care and Pain Medicine, Massachusetts General Hospital, Boston, MA USA; 3 Harvard Medical School, Boston, MA USA; 4 Department of Biostatistics, University of Alabama at Birmingham, Birmingham, AL USA; 5 School of Nursing, School of Medicine, University of Alabama at Birmingham, Birmingham, AL USA; 6 Section of Cardiology, Birmingham Veterans Affairs Medical Center, Birmingham, AL USA

**Keywords:** Cardiovascular biology, Cardiovascular diseases

## Abstract

Cardiovascular disease (CVD) is a leading cause of mortality among pregnant women in the United States (US). This study assessed cardiovascular health (CVH) using the Life’s Essential 8 (LE8) score, which includes sleep, in a nationally representative sample of pregnant women aged 20–44 years without CVD using the 2011–2020 NHANES data. The cohort included an estimated 1.6 million pregnant and 34.5 million non-pregnant women. Pregnant women had lower mean LE8 scores [69.3(1.2) vs. 72.3(0.4)], physical activity [42.7(3.9) vs. 56.2(1.3)], blood lipids [61.8(3.4) vs. 79.4(0.6)], BMI [54.4(3.2) vs. 60.5(1.0)], and diet [43.7(2.5) vs. 43.8(0.7)] than non-pregnant women. They were 51% less likely to have ideal CVH [OR_adj_: 0.49 (95%CI: 0.31–0.77)]. The mean LE8 score in pregnant women was 71.0 (2.2) in 2011–2012 and 66.4 (1.5) in 2017–2020.

## Introduction

Cardiovascular disease (CVD) is the leading contributor to the rising mortality in pregnant women in the United States (US)^1,2^. The high cardiovascular mortality in this otherwise young population may be attributed to the increased burden on the cardiovascular system during pregnancy, which may lead to the rapid progression of subclinical CVD^3^. These pregnancy-induced hemodynamic changes include renin-angiotensin-aldosterone system activation and hormonal fluctuations, which culminate in an increase in blood volume and cardiac output to meet the feto-maternal metabolic demands^3–5^. Furthermore, pregnancy is recognized as a critical developmental phase for the offspring that determines their future risk of CVD^5,6^. Thus, cardiovascular health (CVH) during pregnancy not only determines the risk of CVD in pregnant women but also predicts the CVH of the offspring^7^. However, data on the characterization of CVH using the Life’s Essential 8 (LE8) score during pregnancy are lacking.

In 2022, the American Heart Association (AHA) introduced a new metric to measure CVH, the LE8^8^. In addition to the seven components of the previous metric (Life’s Simple 7 score), the LE8 score recognized sleep as a determinant of CVH^8^. The recognition of sleep as a component of CVH is especially important in pregnant women since pregnancy is thought to be a period of disturbed sleep^9–11^. Furthermore, the multi-dimensionality of sleep as a factor of CVH is highlighted by its association with blood pressure, insulin resistance, obesity, and lipid levels^12–16^. Therefore, it is crucial to incorporate sleep in the characterization of CVH of pregnant women.

The current study applied the LE8 score to women aged between 20 and 44 years in the National Health and Nutrition Examination Survey (NHANES) data between 2011 and 2020 to (1) examine the CVH among pregnant women; (2) assess the mean LE8 score changes in women across the study period; and (3) compare the prevalence of ideal CVH among pregnant and non-pregnant women.

## Results

**Fig. 1:**
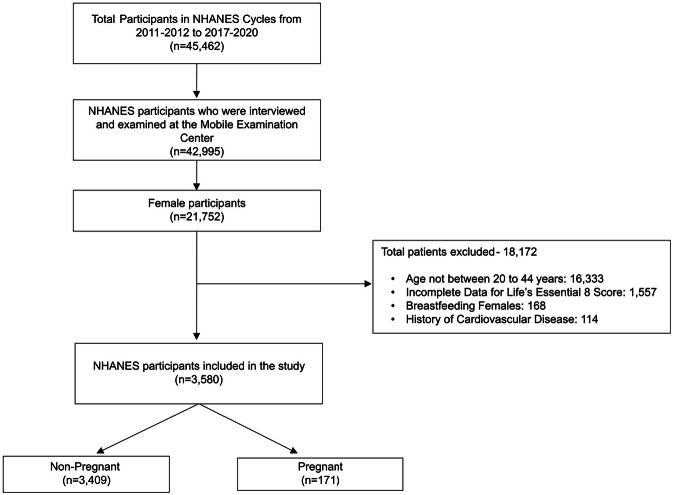
Flowchart of the study population. This figure depicts the study population derivation with stepwise exclusions.

There were 45,462 participants in the NHANES cycles from 2011–2012 to 2017–March 2020. Among these participants, 42,995 individuals underwent physical examination and 21,752 were women. Of the 21,752 women participants, 18,172 individuals were excluded (age <20 years or >44 years: 16,333, breastfeeding women: 168, women with a history of CVD: 114, and individuals with missing data for computation of the LE8 score: 1557). This study included 3580 women, which included 171 pregnant women, representing 1.6 million US women, and 3409 non-pregnant women, representing 34.5 million US women (Fig. 1). The mean LE8 score was [69.3 (1.2)] in pregnant women and [72.3 (0.4)] in non-pregnant women. The blood sugar component of the LE8 score was the highest scoring in non-pregnant women [91.4 (0.4)] and pregnant women [95.3 (1.2)]. The lowest scoring component in non-pregnant women [43.8 (0.7)] and pregnant women [43.7 (2.5)] was the diet score (Table 1). Additionally, the baseline characteristics comparison among women with prevalent cardiovascular events with women without prevalent cardiovascular events was reported (Supplementary Table 3).

**Table 1:** Baseline characteristics and the life’s essential 8 scores of women in the overall population and stratified by pregnancy status in the National Health and Nutrition Examination Survey 2011–2020

Parameter	Overall		Non-pregnant		Pregnant	
	[*n* = 3580 (36,107,619)]		[*n* = 3409 (34,467,961)]		[*n* = 171 (1,639,658)]	
Age	31.3 (25.1, 37.9)		31.6 (25.2, 38.1)		28.3 (24.0, 32.6)	
Education level						
High school or less	28.5 (25.5, 31.5)		28.5 (25.4, 31.6)		29.5 (20.3, 38.6)	
Some college	35.8 (33.3, 38.2)		35.9 (33.3, 38.4)		33.6 (25.4, 41.9)	
College graduate	35.7 (32.1, 39.3)		35.6 (32.0, 39.3)		36.9 (27.4, 46.3)	
Insurance status						
Insured	80.1 (78.1, 82.1)		79.7 (77.7, 81.7)		88.8 (83.7, 93.9)	
Uninsured	19.9 (17.9, 21.9)		20.3 (18.3, 22.3)		11.2 (6.1, 16.3)	
Family poverty income ratio						
≥3.50	36.8 (33.3, 40.2)		36.6 (33.1, 40.1)		40.4 (28.9, 52.0)	
1.30–3.49	35.5 (33.1, 38.0)		35.6 (33.1, 38.1)		33.7 (24.7, 42.7)	
<1.30	27.7 (25.1, 30.3)		27.8 (25.1, 30.4)		25.9 (19.0, 32.7)	
Number of healthcare visits						
None	13.6 (12.2, 15.0)		14.1 (12.7, 15.5)		4.1 (1.0, 7.2)	
1–3	51.4 (49.1, 53.7)		52.3 (50.0, 54.6)		33.1 (22.7, 43.5)	
** ≥4**	35.0 (32.9, 37.1)		33.6 (31.5, 35.7)		62.8 (52.0, 73.6)	
Ethnicity						
White	1183 (33.04%)		1131 (31.59%)		52 (30.41%)	
Black	857 (23.94%)		810 (22.63%)		47 (27.49%)	
Mixed	549 (15.34%)		528 (14.75%)		21 (12.28%)	
Other	991 (27.68%)		940 (26.26%)		51 (29.82%)	

	Median (IQR)	Mean (SE)	Median (IQR)	Mean (SE)	Median (IQR)	Mean (SE)
Essential 8 score	73.0 (61.3, 83.5)	72.2 (0.4)	73.2 (61.3, 83.7)	72.3 (0.4)	67.9 (58.8, 78.9)	69.3 (1.2)
Physical activity score	68.8 (0.0, 93.7)	55.6 (1.3)	71.6 (0.0, 93.8)	56.2 (1.3)	0.0 (0.0,90.5)	42.7 (3.9)
Blood pressure score	86.6 (78.8, 93.3)	87.9 (0.5)	86.5 (77.3, 93.2)	87.6 (0.6)	88.6 (82.9, 94.3)	94.7 (1.7)
Blood lipids score	82.7 (46.8, 91.4)	78.6 (0.6)	83.0 (47.6, 91.5)	79.4 (0.6)	51.6 (24.2, 74.8)	61.8 (3.4)
Blood sugar score	75.6 (63.4, 87.8)	91.5 (0.4)	75.5 (63.2, 87.7)	91.4 (0.4)	78.0 (67.1, 89.0)	95.3 (1.2)
Body mass index score	47.3 (18.0, 79.2)	60.2 (0.9)	48.0 (18.1, 79.6)	60.5 (1.0)	37.8 (17.2, 66.4)	54.4 (3.2)
Smoking score	84.6 (54.2, 92.3)	75.2 (0.8)	84.6 (55.0, 84.6)	75.1 (0.9)	84.9 (43.4, 92.5)	76.4 (4.1)
Sleep score	91.4 (60.1, 95.7)	84.6 (0.5)	91.4 (59.8, 95.7)	84.6 (0.5)	91.1 (71.0, 95.6)	85.6 (1.9)
Diet score	28.1 (6.8, 55.4)	43.8 (0.7)	28.1 (6.8, 55.4)	43.8 (0.7)	28.5 (6.5, 55.8)	43.7 (2.5)

Median (interquartile range), mean (standard error), and percentage (95% CI) have been used to describe data.

### Trends of LE8 score and its components

**Fig. 2:**
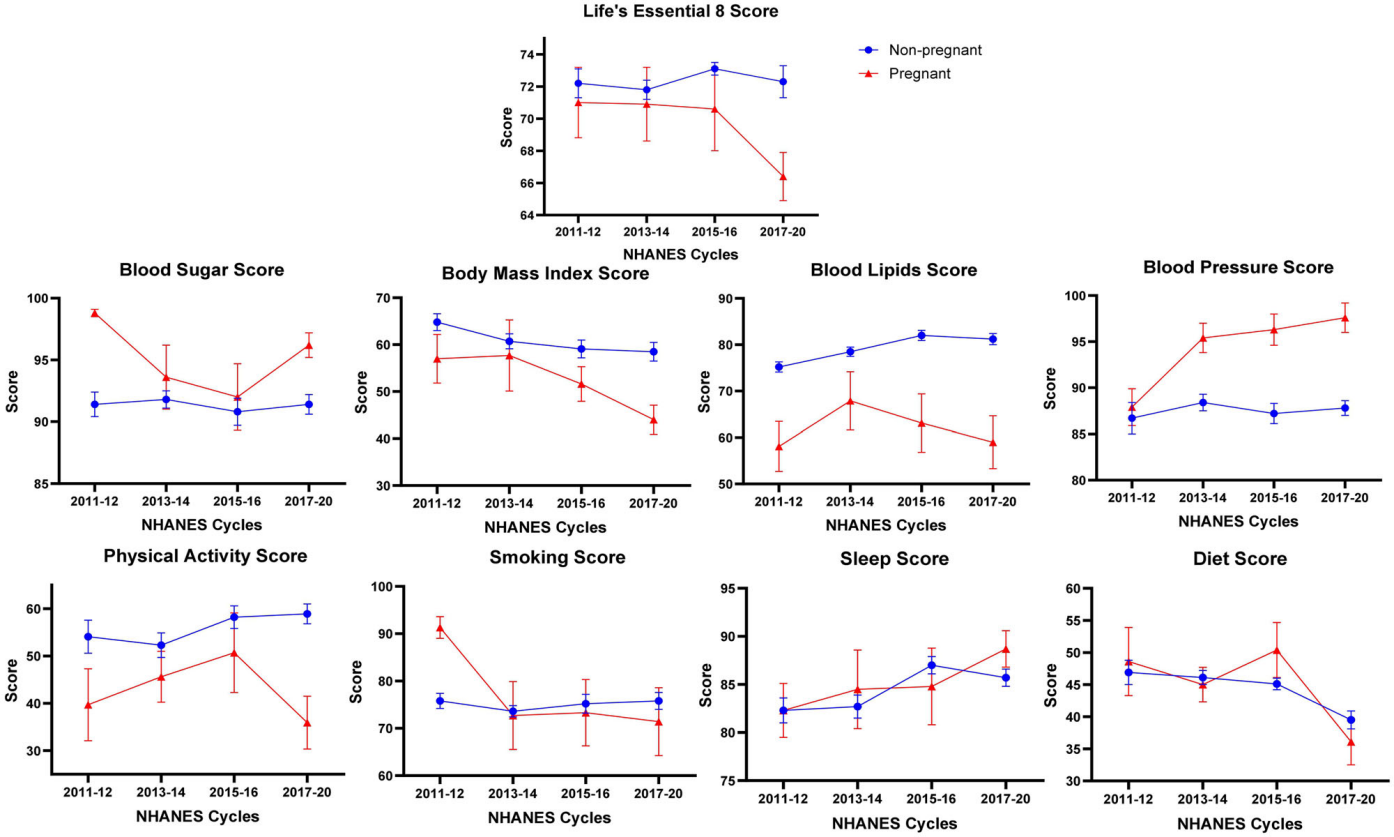
Trends of life’s essential 8 score and its components stratified by pregnancy status in the National Health and Nutrition Examination Cycles 2011–March 2020. This figure depicts the trends of the life’s essential 8 score and its components among women aged between 20 and 44 years stratified by pregnancy status from 2011 to 2020. Pregnant and non-pregnant females have been depicted in red and blue, respectively.

Among pregnant women, the LE8 score was 71.0 (2.2) in 2011–2012 and changed to 66.4 (1.5) in 2017–2020. Additionally, there was a change in the BMI score from 61.3 (5.2) to 46.6 (3.2), smoking score from 91.3 (2.3) to 71.4 (7.2), physical activity from 39.7 (7.6) to 35.9 (5.6), and diet from 48.6 (5.3) to 36.1 (3.6) from the 2011–2012 cycle to the 2017–2020 cycle. The blood pressure score changed from 87.9 (2.0) to 97.6 (1.6), blood lipids from 58.1 (5.4) to 59.0 (5.7), and sleep from 82.3 (2.8) to 88.7 (1.9) from the 2011–2012 cycle to the 2017–2020 cycle (Table 2 and Fig. 2).

**Table 2:** Trends of the Life’s Essential 8 score and its components in pregnant women in the National Health and Nutrition Examination Survey 2011–2020

	2011–2012	2013–2014	2015–2016	2017–2020
Pregnant				
Essential 8 score	71.0 (2.2)	70.9 (2.3)	70.6 (2.6)	66.4 (1.5)
Physical activity score	39.7 (7.6)	45.6 (5.4)	50.7 (8.4)	35.9 (5.6)
Blood pressure score	87.9 (2.0)	95.4 (1.6)	96.3 (1.7)	97.6 (1.6)
Blood lipids score	58.1 (5.4)	67.9 (6.3)	63.1 (6.3)	59.0 (5.7)
Blood sugar score	98.8 (0.3)	93.6 (2.6)	92.0 (2.7)	96.2 (1.0)
Body mass index score	61.3 (5.2)	59.1 (7.3)	54.0 (4.1)	46.6 (3.2)
Smoking score	91.3 (2.3)	72.7 (7.2)	73.3 (7.0)	71.4 (7.2)
Sleep score	82.3 (2.8)	84.5 (4.1)	84.8 (4.0)	88.7 (1.9)
Diet score	48.6 (5.3)	45.0 (2.7)	50.4 (4.3)	36.1 (3.6)

Among non-pregnant women, the LE8 score remained stable between 2011 and 2012 [72.2 (0.9)] and 2017–2020 [72.3 (1.0)] (*P*_linear_: 0.67 and *P*_quadratic_: 0.26). There was an increase in blood lipids [75.2 (1.1) in 2011–2012 to 81.2 (1.2) in 2017–2020; *P*_linear_ < 0.001 and *P*_quadratic_: 0.02] and sleep [82.3 (1.3) in 2011–2012 to 85.7 (0.9) in 2017–2020; *P*_linear_: 0.01] scores. The BMI [64.8 (1.8) in 2011–2012 to 58.5 (2.0) in 2017–2020 (*P*_linear_: 0.001)], and diet [46.9 (1.9) in 2011–2012 to 39.5 (1.4) in 2017–2020 (*P*_linear_: 0.001)] scores decreased across the study period. The physical activity [54.1 (3.5) in 2011–2012 to 58.9 (2.1) in 2017–2020; *P*_linear_: 0.14], blood pressure [86.7 (1.7) in 2011–2012 to 87.8 (0.8) in 2017–2020; *P*_linear_: 0.86], blood sugar [91.4 (1.0) in 2011–2012 to 91.4 (0.8) in 2017–2020; *P*_linear_: 0.51], and smoking [75.8 (1.6) in 2011–2012 to 75.8 (1.8) in 2017–2020; *P*_linear_: 0.48] scores remained stable across the study duration (Table 3 and Fig. 2).

**Table 3:** Trends of the Life’s Essential 8 score and its components in non-pregnant women in the National Health and Nutrition Examination Survey 2011–2020

	2011–2012	2013–2014	2015–2016	2017–2020	Linear *P*_trend_	Quadratic *P*_trend_
Non-pregnant						
Essential 8 score	72.2 (0.9)	71.8 (0.6)	73.1 (0.4)	72.3 (1.0)	0.67	0.26
Physical activity score	54.1 (3.5)	52.3 (2.6)	58.2 (2.4)	58.9 (2.1)	0.14	0.98
Blood pressure score	86.7 (1.7)	88.4 (0.9)	87.2 (1.1)	87.8 (0.8)	0.86	0.48
Blood lipids score	75.2 (1.1)	78.5 (1.0)	82.0 (1.1)	81.2 (1.2)	**<0.001**	**0.02**
Blood sugar score	91.4 (1.0)	91.8 (0.7)	90.8 (1.1)	91.4 (0.8)	0.51	0.89
Body mass index score	64.8 (1.8)	60.7 (1.6)	59.1 (1.9)	58.5 (2.0)	**0.001**	0.42
Smoking score	75.8 (1.6)	73.6 (1.2)	75.2 (2.0)	75.8 (1.8)	0.48	0.63
Sleep score	82.3 (1.3)	82.7 (1.2)	87.0 (0.9)	85.7 (0.9)	**0.01**	0.15
Diet score	46.9 (1.9)	46.1 (1.1)	45.1 (0.9)	39.5 (1.4)	**0.001**	0.08

Bold values represent the results that are significant.

### Odds of ideal LE8 score and its components with pregnancy status

The odds of having an ideal LE8 score were 51% lower in pregnant women compared with non-pregnant women [OR_adj_: 0.49; 95% CI: 0.31–0.77]. The odds of having ideal scores in the physical activity [OR_adj_: 0.47; 95% CI: 0.31–0.71], blood lipids [OR_adj_: 0.39; 95% CI: 0.26–0.58], and BMI [OR_adj_: 0.42; 95% CI: 0.25–0.69] components of the LE8 score were lower in pregnant women compared with non-pregnant women. Pregnant women had higher odds of having an ideal blood pressure score [OR_adj_: 2.07; 95% CI: 1.02–4.22] compared with non-pregnant women. A sensitivity analysis was conducted in which the blood sugar and BMI levels were not included in the computation of the LE8 score, demonstrating that the odds of achieving an ideal LE8 score remained significantly lower in pregnant women compared with non-pregnant women, even after exclusion of these components [OR_adj_: 0.44; 95% CI: 0.28–0.70] (Table 4).

**Table 4:** Odds ratio of ideal Life’s Essential 8 score and its components in the women and stratified by pregnancy status in the National Health and Nutrition Examination Survey 2011–2020

	Life’s Essential 8 score	Diet	Physical activity	Blood pressure	Blood lipids	Blood sugar	Body mass index	Smoking	Sleep	Life’s essential 8 score sensitivity analysis*
Non pregnant	Reference									
Pregnant	**0.49 (0.31–0.77)**	1.13 (0.77**–**1.67)	**0.47 (0.31–0.71)**	**2.07 (1.02–4.22)**	**0.39 (0.26–0.58)**	1.68 (0.95**–**2.96)	**0.42 (0.25–0.69)**	0.79 (0.43**–**1.24)	1.29 (0.88**–**1.90)	**0.44 (0.28–0.70)**

Odds ratios (95% confidence interval) of ideal levels (≥80) of the Life’s Essential 8 score and its components as the outcome of interest, with non-ideal levels (0–80) as a reference, have been presented. Non-pregnant women were taken as the reference population.

*Sensitivity analysis in which LE8 was computed without the blood sugar and BMI scores.

Bold values represent the results that are significant.

### Age-stratified analysis

Among pregnant women, the LE8 score was the highest in the 25–34 years age group [72.0 (1.3)] compared with the 20–24 years [66.8 (1.9)] and 35–44 years [65.2 (2.8)] age groups, although not statistically significant (P: 0.09). The higher LE8 scores in the 25–34 years age group was primarily driven by the smoking (P: 0.03) and sleep (P: 0.04) scores. Additionally, the sensitivity analysis wherein the LE8 score was computed without the blood sugar and BMI components demonstrated a similar pattern with the LE8 score highest in the 25–34 years age group [70.5 (1.5)] compared with the 20–24 years [64.5 (2.1)] and 35–44 years [62.8 (3.3)] age groups, although not statistically significant (P: 0.11). (Table 5)

**Table 5:** Components of LE8 stratified by age in pregnant and non-pregnant women.

Pregnant	20–24 years	25–34 years	35–44 years	*p*-value
	[*n* = 45 (413,388)]	[*n* = 94 (902,879)]	[*n* = 32(323,391)]	
	Mean (SE)	Mean (SE)	Mean (SE)	
Essential 8 score	66.8 (1.9)	72.0 (1.3)	65.2 (2.8)	0.085
Physical activity score	40.4 (6.0)	48.8 (4.8)	28.7 (5.7)	0.43
Blood pressure score	99.2 (0.6)	94.1 (1.2)	90.7 (2.4)	0.67
Blood lipids score	60.0 (5.4)	66.4 (4.7)	51.5 (6.3)	0.09
Blood sugar score	97.0 (1.0)	95.8 (1.0)	91.7 (3.2)	0.9
Body mass index score	50.3 (4.0)	56.6 (4.3)	53.1 (5.9)	0.41
Smoking score	65.5 (6.7)	82.3 (3.0)	73.8 (9.5)	**0.03**
Sleep score	79.0 (4.3)	90.8 (1.4)	79.7 (4.0)	**0.04**
Diet score	43.0 (4.7)	40.9 (2.6)	52.5 (6.2)	**0.007**
Essential 8 score^a^ sensitivity analysis	64.5 (2.1)	70.5 (1.5)	62.8 (3.3)	0.113

Non-pregnant	20–24 Years	25–34 Years	35–44 Years	*p*-value
	[*n* = 661 (6,908,487)]	[*n* = 1,319 (13,436,647)	[*n* = 1,429 (14,122,827)]]	
	Mean (SE)	Mean (SE)	Mean (SE)	
Essential 8 score	75.4 (0.8)	73.4 (0.6)	69.8 (0.5)	0.06
Physical activity score	58.2 (2.5)	59.1 (1.6)	52.4 (1.9)	0.77
Blood pressure score	94.3 (1.0)	90.7 (0.8)	81.3 (1.0)	0.44
Blood lipids score	85.2 (1.0)	81.4 (0.9)	74.7 (1.0)	0.25
Blood sugar score	96.3 (0.5)	93.4 (0.6)	87.0 (0.8)	0.9
Body mass index score	68.8 (2.0)	59.9 (1.5)	57.0 (1.2)	**0.002**
Smoking score	77.2 (2.0)	74.8 (1.2)	74.5 (1.2)	0.25
Sleep score	82.5 (1.3)	85.3 (0.8)	84.8 (0.8)	0.89
Diet score	40.9 (1.4)	42.7 (1.0)	46.3 (0.9)	0.64

^a^Sensitivity analysis in which LE8 was computed without the blood sugar and BMI scores.

Bold values represent the results that are significant.

Among non-pregnant women, the LE8 score was the highest in the 20–24 years age group [75.4 (0.8)] compared with the 25–34 years [73.4 (0.6)] and 35–44 years [69.8 (0.5)] age groups, although statistical significance was not achieved (P: 0.06). The variation in the LE8 score by age was primarily driven by the BMI score, which was the highest in the 20–24 years age group [68.8 (2.0)] compared with the 25–34 years [59.9 (1.5)] and 35–44 years [57.0 (1.2)] age groups (*P*_trend_: 0.001). (Table 5)

## Discussion

This study utilized population-level data from the NHANES to assess and compare CVH in pregnant and non-pregnant women aged 20–44 years, utilizing the LE8 score. The results revealed that pregnant women exhibit lower overall CVH compared to their non-pregnant counterparts, primarily driven by poorer scores in physical activity and blood lipids. On examining the trends of CVH and its components between 2011 and 2020, the LE8 score remained stable in non-pregnant women. A decrease in BMI scores was noted in non-pregnant women. Additionally, an improvement in the sleep and blood lipid components was noted across the study period among non-pregnant women. The sample size of pregnant women was insufficient to reliably analyze secular trends; therefore, only mean descriptive values across the cycles have been reported. Furthermore, pregnant women demonstrated significantly lower odds of achieving ideal CVH, physical activity, blood lipids, and BMI, but had a higher likelihood of maintaining ideal blood pressure levels compared to non-pregnant women. Overall, pregnant women were half as likely as their non-pregnant counterparts to achieve ideal CVH, with consistent results in the sensitivity analysis wherein the LE8 was computed without the sugar and BMI components.

The lower CVH in pregnant women is concerning from a public health perspective, especially since pregnancy is a critical period that can influence both maternal and fetal health outcomes. The numerical decrease in the mean LE8 score was accompanied by a substantial decrease in the mean BMI score across the study time frame. A consistent decline in dietary scores may have contributed to the increased BMI seen over the study period, formal statistical testing of these trends was not performed due to the limited number of pregnant women per cycle. However, this trend aligns with the global patterns being seen today, as obesity rates have risen worldwide^17^. Previous studies have found that purchases of processed foods have been associated with overweight/obesity^18^. Pregnant women who are obese before or during pregnancy have an increased risk of CVD and are more susceptible to developing adverse pregnancy outcomes such as preeclampsia, gestational diabetes, and high blood pressure^19^. There are various cardiac adaptations that pregnant women undergo during pregnancy, including increases in stroke volume and decreases in vascular resistance^17^. However, as women approach and enter advanced maternal age (35 years and older), they experience a loss in vascular compliance and endothelial function, making them less likely to adapt to the physiologic cardiac changes observed during pregnancy, leading to less favorable CVH and pregnancy outcomes^20,21^. Adolescent and young adult women who are pregnant are less likely to seek prenatal care, thus increasing their risk for adverse health conditions. These trends potentially explain why CVH was most favorable in the 25–34 aged subgroup, compared to the 20–24 and 35–44 aged subgroups.

Notably, although pregnant women had lower odds of achieving ideal CVH as per the LE8 score and the sensitivity analysis (wherein the LE8 score was computed without the blood sugar and BMI components), they showed a significantly higher likelihood of having ideal blood pressure levels and had no difference in the blood sugar levels. This finding is somewhat unexpected given the known risk of gestational diabetes mellitus and hypertensive disorders in pregnancy, including preeclampsia^3^. However, this result should be interpreted with caution. NHANES does not include gestational age data, so we were unable to differentiate between early and late pregnancy, when preeclampsia and gestational diabetes mellitus typically develop. In addition, the age distribution may partially explain this finding: pregnant women in our study were younger (median age 28.3 years, IQR: 24.0–32.6) than non-pregnant women (median age 31.6 years, IQR: 25.2–38.1). Since blood pressure tends to increase with age, this younger profile likely contributed to the higher odds of ideal blood pressure among pregnant participants. Blood sugar was assessed using fasting glucose or HbA1c, which may not adequately capture pregnancy-related insulin resistance, particularly in the absence of oral glucose tolerance testing, which was not performed in NHANES^22^. Sleep was evaluated based solely on duration, without accounting for common pregnancy-related disturbances such as fragmentation or poor sleep quality^23^. Changes in smoking and diet during pregnancy may also have been underrepresented, as only modest score improvements are assigned for smoking cessation, and dietary intake was based on single 24-h recalls, which may not reflect habitual intake and are subject to reporting bias. These measurement limitations may have contributed to the lack of significant associations observed in these CVH components among pregnant women.

Previous work assessing CVH using LS7 in pregnant women aged 20–44 from 1999 to 2012 also found that CVH was worse in pregnant women compared to non-pregnant^24^. This study noted the lower CVH score in pregnant women could be attributed to less favorable physical activity and total cholesterol levels, which aligned with the results we obtained using LE8 scores. However, one point of difference comes in the classification of CVH scores in these patients. Compared to our findings, the previous work using LS7 scores found that 34.8% had poor CVH, 60.6% had moderate CVH, and 4.6% had ideal CVH^24^. Thus, while the individual component scores shared similar findings, the overall CVH score classification showed a difference in using LS7 versus LE8 scores, as more individuals were classified as having ideal CVH in LE8 than LS7. This difference can partially be explained by the point system scales used as LS7 is assessed on a 14-point scale while LE8 is on a 100-point scale and allows for greater score delineation. The differences present where the LE8 score uses the HEI 2015 for diet score and the LS7 was based on the diet guidelines given by AHA. Furthermore, the LE8 considers both first and secondhand exposure for nicotine, whereas the LS7 score only focuses on firsthand cigarette smoking^25,26^.

The public health implications of these findings are substantial. Poor physical activity, unhealthy lipid profiles, and elevated BMI among pregnant women are significant risk factors for adverse cardiovascular outcomes. The lack of physical exercise can lead to various cardiovascular problems, including high blood pressure, weight gain, and higher cholesterol levels^27^. Elevated lipid profiles provide insight into how higher levels of cholesterol and triglycerides can contribute to the development of atherosclerosis and have been shown to increase the risk of preeclampsia and gestational diabetes^28,29^. Until recently, statins were contraindicated in pregnancy due to concerns about potential teratogenic effects, and guidelines continue to recommend caution about their use^30^. Therefore, the suboptimal lipid control in pregnant women may stem from the lack of use of statins. Bile acid sequestrants, the most commonly prescribed lipid-lowering agents during pregnancy, offer only modest reductions in lipid levels compared to statins^30^. This highlights a critical gap in lipid management during pregnancy and underscores the need for safer, more effective alternatives and clearer clinical guidance. High BMI levels during pregnancy have been linked to developing a higher risk for CVD later in life for both the mother and the offspring^31^. Measures that can be taken to improve CVH include enhancing physical activity, improving diet, regular health checkups, and educational and behavioral interventions^32,33^. Educational and behavioral interventions targeting these areas could improve both maternal and fetal health outcomes. Programs that promote healthy lifestyle choices, provide accessible prenatal care, and raise awareness about the risks of obesity and CVD during pregnancy are crucial for improving CVH in this population. While physicians have effectively managed blood pressure in pregnant women, there is a notable discrepancy in the diet and weight aspects of managing CVH. Prenatal visits present as a great opportunity to address the deteriorating dietary and exercise trends among pregnant women and should place a greater emphasis on tailoring an individualized nutritional and exercise plan to address the deficiencies in these crucial aspects of CVH. Additionally, this study could not formally assess secular trends across the NHANES cycles due to limitations in sample size, reflecting the underrepresentation of this cohort. Efforts geared to better understand and quantify the trends of CVH among pregnant women should be implemented at a national level. Lastly, currently, the LE8 score does not factor in gestational age-specific thresholds despite including metrics such as BMI and blood sugars, which vary considerably among pregnant women according to gestational age. Incorporating these factors in the future iterations of CVH scores would help improve the generalizability of these scores among pregnant women, and, accordingly, plan high-yield policy changes and public health interventions.

The study had a few limitations. First, the NHANES lacked data on the gestational age which did not allow the application of trimester-specific definitions for health factors. Especially, blood sugars and BMI demonstrate considerable variations with gestational age and the results should be interpreted with caution. Second, the oral glucose tolerance test is commonly used to detect the presence of gestational diabetes mellitus. However, the NHANES does not include an oral glucose tolerance test using pregnancy specific modifications. Third, the cross-sectional nature of the NHANES does not permit the inference of causality. Fourth, the NHANES uses self-reported data for several components, which are susceptible to recall bias and measurement errors. Fifth, the exclusion of women with missing data may increase the variance in the estimates of CVH presented in the study. Sixth, the NHANES discontinued oversampling of pregnant women after the 2007–2008 cycle. Therefore, even after including data from the NHANES cycles between 2010 and 2020, the sample size of pregnant women was limited. Accordingly, secular trend analysis for pregnant women was not performed.

This nationwide population-based analyses demonstrated CVH in pregnant women was lower than non-pregnant women, which was mainly contributed by the physical activity and blood lipids components. Pregnant women were only half as likely to have ideal CVH and ideal levels of physical activity, blood lipids, and BMI compared with non-pregnant women. Across the study period, the mean LE8 scores in pregnant women did not vary considerably. The current study highlights the need to improve CVH among pregnant women by targeting specific components of CVH, including physical activity, blood lipids, and BMI to improve maternal and offspring well-being.

## Methods

### Data source

This study combined 4 NHANES cycles from 2011–2012 to 2017–2020. The NHANES is a cross-sectional survey conducted by the Center for Disease Control and Prevention (CDC) and the National Center for Health Statistics (NCHS) every two years to assess the health and nutritional status of a sample population utilizing a complex multistage sampling design^34–39^. All participants underwent a home interview during which data on sleep, physical activity, diet, medical conditions such as hypertension, smoking, and medication use were collected^34^. Individuals who consented to a clinical examination were invited to a mobile examination center where a detailed physical examination, vital sign measurement, anthropometry, and blood collection for laboratory testing were done^34^. Informed consent was collected before the home interview and physical examination. Ethical oversight for this study was provided by the Institutional Review Board at the University of Alabama at Birmingham.

### Study participants

This study included women aged 20–44 years. Human chorionic gonadotropin (hCG) (Beckman Coulter) urine pregnancy tests were used to identify pregnant women^40^. This study excluded women who did not undergo the physical examination, individuals with missing data for the computation of the LE8 score, and those with self-reported prevalent CVD (coronary artery disease, heart failure, angina, heart attack, and stroke)^41^. The study population was further stratified by age 20–24 years, 25–34 years, and 35–44 years.

### Life’s essential 8 score computation

The LE8 score is composed of four health behaviors and four health factors. Health behaviors include smoking, physical activity, sleep, and diet^8^. The health factors include blood pressure, blood glucose, blood lipids, and body mass index (BMI)^8^. Each metric is scored from 0 to 100 and the LE8 score is calculated by taking the mean of the eight components. The scoring scheme for the components of the LE8 score has been described in Supplementary Table 1^39^. Ideal CVH was defined as an LE8 score of ≥80.

The tobacco use questionnaire was utilized to collect data on self-reported smoking status, use of electronic nicotine devices (vape, cigars, pipes, hookahs) in the past 5 days, and the years since smoking cessation. The household smoker’s questionnaire was used to determine the exposure to secondhand smoke.

Physical activity was quantified based on the self-reported intensity (intense or moderate effort) and the self-reported frequency (number of times in the week and hours per day). Vigorous physical activity was described as strenuous exercise or heavy sweating leading to a large increase in breathing. Moderate physical exercise was described as exercise leading to a small increase in breathing^42^.

The Dietary Approach to Stop Hypertension (DASH) was utilized to calculate scores using data from two 24-h dietary recalls^43^. The score consisted of nine calorie-indexed values for different food groups, including total fat, saturated fat, protein, fiber, cholesterol, magnesium, potassium, calcium, and sodium^44^. DASH targets were assigned ideal and intermediate values at 1.0 and 0.5, respectively (Supplementary Table 2)^39,44^.

The sleep disorders questionnaire was used to determine the sleep duration in a day^45^.

Blood pressure measurements were taken as an average of three readings taken after 5 min of rest. The mean of three measurements was used to calculate both systolic and diastolic values. If three readings were not taken, the first reading was used for the systolic and diastolic values^39^. The home interview form was used to determine the usage of anti-hypertensive medications.

BMI was calculated from the height (meters) and weight (kilograms) measured during the physical examination.

Blood samples collected during the physical examination were used to determine fasting blood glucose (hexokinase-based enzyme assay), cholesterol (enzymatic assay), and HbA_1_C levels (high-performance liquid chromatography). HDL cholesterol levels were subtracted from total cholesterol levels to determine non-HDL levels. The home interview form was used to determine lipid-lowering medication and medications for diabetes, such as oral hypoglycemic agents and insulin.

The study used the following covariates: education level (high school or less, some college, or college graduate), insurance status (insured or uninsured), family poverty income ratio (<1.30 [low socioeconomic status], 1.30–3.49 [moderate socioeconomic status], ≥3.50 [high socioeconomic status]) number of healthcare visits in a year (none, 1 to 3, or 4 or more), and ethnicity^39^.

### Sensitivity analysis

The blood sugars and the BMI scores vary considerably with gestational age, and the NHANES stopped reporting the month of pregnancy from 2013 to 2014 onward cycles^24,46,47^. Hence, a sensitivity analysis was conducted by computing the Life’s Essential 8 score without the blood sugar and the BMI components. The score was computed as the mean of the remaining six components, namely the diet, physical activity, smoking status, blood pressure, blood lipids, and sleep scores. The score for the sensitivity analyses was also reported on a scale of 0–100.

### Statistical analysis

All analyses were conducted using SAS 9.4 (Cary, NC)^12^. The SURVEY procedures in SAS were used to take into consideration the multistage sample design of the NHANES data as recommended by NCHS^36^^,^^45^^,^^48^. The physical examination sample weights were used for analysis. These weights were adjusted to account for the combining cycles from 2011 to 2020 as recommended^34^. The analyses were conducted in the overall population and stratified by age. Descriptive values of LE8 and its components were estimated across 4 cycles (2011–2012, 2013–2014, 2015–2016, and 2017–March 2020). The linear and quadratic p-values for the trends in pregnant women were not reported due to the small sample size per cycle, and only descriptive estimates are presented. Multivariable adjusted logistic regression models were used to examine the odds of ideal LE8 and component scores using non-pregnant women as the reference group. Statistical significance was set at a two-sided *p*-value of <0.05.

## Supplementary information

Supplementary Tables

## Data Availability

The National Health and Nutrition Examination Survey is a survey conducted biennially by the NCHS and CDC to assess the health status of the civilian non-institutionalized population of the United States. The data is made publicly available and was downloaded from https://www.cdc.gov/nchs/nhanes/Default.aspx.

## Code availability

The code for the analysis will be made available upon request. All analyses were conducted using SAS 9.4 (Cary, NC).
