# Supplementary material for: Cardiovascular health in pregnancy according to Life’s Essential 8 score
Source: NPJ Cardiovasc Health. 2026 Apr 1;3:18. doi: 10.1038/s44325-026-00117-6 (PMC13043774; doi:10.1038/s44325-026-00117-6)
Supplement: Supplementary file 1 — Supplementary Tables [file 44325_2026_117_MOESM1_ESM.pdf]

Supplementary Table 1: Scoring Scheme for the Life's Essential 8 Score Components

Health Factors		Health Behaviors	
**Blood Pressure***	**Points**	**Diet**<br>**Quantile of Self-Reported Adherence to DASH Diet**	**Points**
Systolic BP <120 mmHg and Diastolic BP <80 mmHg	100	≥95th percentile	100
Systolic BP 120-129 mmHg and Diastolic BP <80 mmHg	75	75th-94th percentile	80
Systolic BP 130-139 mmHg or Diastolic BP 80-89 mmHg	50	50th-74th percentile	50
Systolic BP 140-159 mmHg or Diastolic BP 90-99 mmHg	25	25th-49th percentile	25
Systolic BP ≥160 mmHg or Diastolic BP ≥100 mmHg	0	1st-24th percentile	0
**Non-HDL Cholesterol***		**Physical Activity**<br>**Self-Reported Minutes of Moderate or Vigorous Intensity Activity per Week**	
<130 mg/dL	100	≥150 minutes	100
130-159 mg/dL	60	120-149 minutes	90
160-189 mg/dL	40	90-119 minutes	80
190-219 mg/dL	20	60-89 minutes	60
≥220 mg/dL	0	30-59 minutes	40
**Blood Glucose**<br>**Glycosylated Hemoglobin A$_1$C**		1-29 minutes	20
Fasting blood glucose <100 mg/dL or HbA$_1$C <5.7 gm% with no history of diabetes mellitus	100	0 minutes	0
Fasting blood glucose 100-125 mg/dL or HbA$_1$C 5.7-6.5 gm% with no history of diabetes mellitus	60	**Smoking** #<br>**Self-Reported Cigarette Smoking**	
HbA$_1$C <7.0 gm% with diabetes	40	Never smoked	100
HbA$_1$C 7.0-7.9 gm%	30	Former smoker, quit ≥5 years ago	75
HbA$_1$C 8.0-8.9 gm%	20	Former smoker, quit 1-<5 years ago	50
HbA$_1$C 9.0-9.9 gm%	10	Former smoker, quit <1 year ago<br>Currently using e-cigarettes	25
HbA$_1$C ≥10.0 gm%	0	Current smoker	0
**Body Mass Index**		**Sleep**<br>**Self-Reported Average Hours of Sleep per Night**	
		7- <9 hours	100
<25.0 kg/m^2	100	9- <10 hours	90
25-29.9 kg/m^2	70	6- <7 hours	70
30-34.9 kg/m^2	30	5- <6 or ≥10 hours	40
35-39.9 kg/m^2	15	4- <5 hours	20
≥40 kg/m^2	0	<4 hours	0

Abbreviations: DBP: diastolic blood pressure, HbA$_1$C: glycosylated hemoglobin A$_1$C, kg/m^2: kilograms per meter squared, mmHg: millimeters of mercury, SBP: systolic blood pressure, mg/dL: milligram per deciliter. *20 point subtracted if treated levels. #20 points subtracted if living with an active indoor smoker.

Supplementary Table 2: Scoring Scheme for the Life's Essential 8 Score Components

Components	DASH Score Target	Intermediate Target
Saturated Fat	6% of daily energy	11% of daily energy
Total Fat	27% of daily energy	32 % of daily energy
Protein	18% of daily energy	16.5% of daily energy
Cholesterol*	71.4 mg	107.1 mg
Fiber*	14.8 g	9.5 g
Magnesium*	238 mg	158 mg
Calcium*	590 mg	402 mg
Potassium*	2,238 mg	1,534 mg
Sodium*	1,143 mg	1,286 mg

*Indexed to per 1,000 kcal

Supplementary Table 3: Baseline Characteristics Comparison of Women with Prevalent Cardiovascular Outcomes with Women without Prevalent Cardiovascular Outcomes.

Parameter	Overall [n=3,580 (36,107,619)]		CVD Subgroups [n=114 (1,014,891)]	
**Age***	31.3 (25.1, 37.9)		28.3 (24.0, 32.6)	
**Pregnant**	171 (4.8)		2 (1.8)	
**Education level**				
**High School or Less**	28.5 (25.5, 31.5)		43.1 (35.2, 51.0)	
**Some College**	35.8 (33.3, 38.2)		35.4 (28.9, 41.9)	
**College Graduate**	35.7 (32.1, 39.3)		21.6 (14.3, 28.8)	
**Insurance Status**				
**Insured**	80.1 (78.1, 82.1)		84.0 (75.6. 92.4)	
**Uninsured**	19.9 (17.9, 21.9)		16.0 (7.6, 24.4)	
**Family Poverty Income Ratio**				
**>=3.50**	36.8 (33.3, 40.2)		11.3 (4.5, 20.0)	
**1.30-3.49**	35.5 (33.1, 38.0)		33.0 (22.6, 43.4)	
**<1.30**	27.7 (25.1, 30.3)		55.8 (46.1, 65.5)	
**Number of Healthcare Visits**				
**None**	13.6 (12.2, 15.0)		13.5 (5.7, 21.3)	
**1 to 3**	51.4 (49.1, 53.7)		26.3 (16.5, 36.1)	
**>=4**	35.0 (32.9, 37.1)		60.2 (48.7, 71.6)	
	**Median (IQR)**	**Mean (SE)**	**Median (IQR)**	**Mean (SE)**
**Essential 8 Score**	73.0 (61.3, 83.5)	72.2 (0.4)	54.8 (46.0, 67.1)	55.7 (1.1)
**Physical Activity Score**	68.8 (0.0, 93.7)	55.6 (1.3)	0.0 (0.0, 92.7)	42.6 (4.2)
**Blood Pressure Score**	86.6 (78.8, 93.3)	87.9 (0.5)	78.2 (41.8, 89.3)	71.0 (3.3)
**Blood Lipids Score**	82.7 (46.8, 91.4)	78.6 (0.6)	71.8 (45.3, 88.9)	75.2 (3.0)
**Blood Sugar Score**	75.6 (63.4, 87.8)	91.5 (0.4)	65.5 (42.1, 82.7)	75.6 (3.0)
**Body Mass Index Score**	47.3 (18.0, 79.2)	60.2 (0.9)	28.2 (4.1, 69.0)	46.8 (3.5)
**Smoking Score**	84.6 (54.2, 92.3)	75.2 (0.8)	60.9 (0.0, 88.2)	53.1 (4.6)
**Sleep Score**	91.4 (60.1, 95.7)	84.6 (0.5)	64.3 (33.0, 93.7)	70.7 (2.8)
**Diet Score**	28.1 (6.8, 55.4)	43.8 (0.7)	20.7 (0.0, 42.0)	34.5 (2.6)
